# Fractional Flow Reserve from Coronary CT: Evidence, Applications, and Future Directions

**DOI:** 10.3390/jcdd12080279

**Published:** 2025-07-22

**Authors:** Arta Kasaeian, Mohadese Ahmadzade, Taylor Hoffman, Mohammad Ghasemi-Rad, Anoop Padoor Ayyappan

**Affiliations:** 1 Baylor College of Medicine, Houston, TX 77030, USA; arta.kasaeian@bcm.edu (A.K.); taylor.hoffman@bcm.edu (T.H.); 2 Department of Radiology, Division of Vascular and Interventional Radiology, Baylor College of Medicine, Houston, TX 77030, USA; mohadese.ahmadzade@bcm.edu; 3 Department of Radiology, Division of Cardiothoracic Imaging, Baylor College of Medicine, Houston, TX 77030, USA

**Keywords:** fractional flow reserve, coronary CT angiography, computational fluid dynamics, coronary artery disease, FFR-CT

## Abstract

Coronary computed tomography angiography (CCTA) has emerged as the leading noninvasive imaging modality for the assessment of coronary artery disease (CAD), offering high-resolution visualization of the coronary anatomy and plaque characterization. The development of fractional flow reserve derived from CCTA (FFR-CT) has further transformed the diagnostic landscape by enabling the simultaneous evaluation of both anatomical stenosis and lesion-specific ischemia. FFR-CT has demonstrated diagnostic accuracy comparable to invasive FFR. The combined use of CCTA and FFR-CT is now pivotal in a broad range of clinical scenarios, including the evaluation of stable and acute chest pain, assessment of high-risk and complex plaque features, and preoperative planning. As evidence continues to mount, CCTA and FFR-CT are positioned to become the primary gatekeepers to the cardiac catheterization laboratory, potentially reducing the number of unnecessary invasive procedures. This review highlights the growing clinical utility of FFR-CT, its integration with advanced plaque imaging, and the future potential of these technologies in redefining the management of CAD, while also acknowledging current limitations, including image quality requirements, cost, and access.

## 1. Introduction

Coronary artery disease (CAD) is the leading cause of morbidity and mortality globally, accounting for approximately 17.8 million deaths each year and affecting about 1 in 20 adults in the United States alone [1]. As the prevalence of CAD continues to rise, there is a growing need for accurate and personalized ways to diagnose it as early as possible. Traditional methods for evaluating CAD, including clinical risk scoring, functional stress testing, and invasive coronary angiography (ICA), have long been the standard of care. However, each comes with notable limitations. ICA, while considered the gold standard, is invasive, expensive, and subject to interobserver variability. More importantly, it often fails to capture the true hemodynamic impact of coronary lesions. Visual assessment of stenosis severity can overlook pathophysiologic elements like collateral circulation or plaque morphology, potentially leading to overtreatment or underestimation of risk [2]. As the precision of CAD diagnostics has improved, the focus has shifted beyond simply identifying luminal stenosis. There is now greater emphasis on understanding the specific lesion site, the extent of luminal narrowing, composition of arterial plaques, and the presence of downstream ischemia and impaired myocardial perfusion [3]. This comprehensive approach is helping to improve risk stratification and guide more personalized treatment strategies.

Against this backdrop, coronary computed tomography angiography (CCTA) has emerged as a powerful, non-invasive tool for assessing CAD. Recent technological innovations have significantly enhanced the spatial and temporal resolution of CCTA while reducing radiation exposure. CCTA has a sensitivity of up to 99% and a negative predictive value of 97%, making it highly effective for excluding significant coronary artery disease in low- to intermediate-risk patients [4]. Current evidence supports a Class I, Level A recommendation for cardiac CT in both stable and acute chest pain, as outlined in the 2021 AHA–ACC Chest Pain Guidelines [5]. Specifically, CCTA is recommended (Class I) for intermediate-risk patients, while FFR-CT carries a Class IIa recommendation for evaluating 40–90% stenosis in proximal or mid-coronary arteries. In the SCOT-HEART trial, 23% of patients who received cardiac CT had their treatment changed within 6 weeks, compared to only 5% with standard care, showing that cardiac CT results can significantly impact individual treatment decisions [6]. The DISCHARGE trial, involving patients with stable chest pain and intermediate CAD risk, found that cardiac CT and invasive angiography had similar outcomes over 3.5 years, but cardiac CT led to fewer major procedural complications [7].

While CCTA has proven valuable for visualizing coronary anatomy, its limitation to anatomical assessment alone has driven the development of CT-derived fractional flow reserve (FFR-CT), an innovation that integrates both anatomical and functional insights. This advancement enhances the ability to evaluate the physiological significance of coronary lesions, improving diagnostic accuracy and guiding more effective treatment strategies. This review explores the role of FFR-CT and its potential to impact prognosis and reshape clinical practice in CAD.

## 2. Fractional Flow Reserve

Fractional flow reserve (FFR) is a physiological index developed in the early 1990s to assess the hemodynamic significance of coronary artery stenoses. In 1993, Dr. Nico H. J. Pijls and Dr. Bernard De Bruyne introduced FFR as a method to quantify the pressure gradient across a coronary lesion during conditions of maximal hyperemia [8,9]. This metric, defined as the ratio of distal coronary pressure to aortic pressure under maximal blood flow, enables a functional assessment of coronary stenoses that complements traditional anatomical imaging. The introduction of FFR marked a significant advancement in interventional cardiology by enhancing the precision of percutaneous coronary intervention (PCI), improving clinical decision-making, and leading to better patient outcomes.

## 3. Clinical Validation of Invasive FFR

### 3.1. The FAME Study: FFR-Guided PCI in Multivessel Disease

The FAME (Fractional Flow Reserve Versus Angiography for Multivessel Evaluation) study evaluated the clinical benefit of FFR-guided PCI in patients with multivessel CAD [10]. In this randomized trial involving 1005 patients, participants were assigned to PCI guided either by angiography alone or by FFR measurements. Lesions with FFR ≤ 0.80 were treated in the FFR group, whereas all angiographically significant lesions were stented in the other group. Although both groups had a similar number of target lesions, fewer stents were implanted in the FFR-guided group (1.9 vs. 2.7; *p* < 0.001). After two years, the FFR group had significantly lower rates of death or myocardial infarction (8.4% vs. 12.9%; *p* = 0.02). Lesions deferred based on an FFR > 0.80 showed very low adverse-event rates. These findings support FFR’s critical role in safely guiding PCI and improving long-term outcomes in multivessel CAD.

### 3.2. The FAME 2 Trial: FFR-Guided PCI vs. Medical Therapy

The FAME 2 study demonstrated that in patients with stable CAD and reduced fractional flow reserve (FFR ≤ 0.80), percutaneous coronary intervention (PCI) plus medical therapy (MT) significantly improved clinical outcomes, compared to MT alone [11]. Over three years, PCI reduced major adverse cardiac events (10.1% vs. 22.0%), primarily by lowering urgent revascularization rates. Death and myocardial infarction rates were also lower, though this decline was not statistically significant. Patients undergoing PCI experienced less severe angina and sustained quality-of-life improvements throughout follow-up. While initial costs were higher for PCI, long-term expenses were comparable to MT alone. The study supports FFR-guided PCI as a superior strategy over MT in stable coronary disease, enhancing survival, reducing cardiac events, and improving patient quality of life without increased long-term costs.

## 4. Emergence of Non-Invasive Coronary Physiology Assessment

Despite the advantages of invasive FFR, the need for pressure wires and pharmacologic agents such as adenosine introduces patient discomfort, procedural risk, and cost. These limitations spurred the development of non-invasive alternatives, particularly leveraging advancements in CCTA and computational modeling.

FFR-CT integrates the anatomical detail provided by CCTA with the physiological insights of traditional FFR, offering a comprehensive, non-invasive assessment of CAD. This approach eliminates the need for pharmacologic stress agents, such as adenosine, and obviates the use of invasive pressure wires. As a result, FFR-CT enhances patient comfort and streamlines the diagnostic process without compromising clinical accuracy.

## 5. Principles and Technology Behind FFR-CT

FFR-CT uses standard CCTA datasets combined with computational fluid dynamics to model coronary blood flow and evaluate the hemodynamic significance of coronary lesions. Traditionally, FFR is measured invasively using pressure wire and pharmacologic agents like adenosine. In contrast, FFR-CT estimates this value non-invasively by modeling blood flow as a Newtonian fluid governed by the Navier–Stokes equations [12]. FFR-CT estimates blood flow and pressure in coronary arteries non-invasively by applying computational fluid dynamics to CCTA images. Total coronary flow at rest is first estimated using heart size and mass as key parameters. Microvascular resistance is then assumed to be inversely related to the size of the epicardial arteries. Because the coronary response to vasodilators is predictable, maximal blood flow can be simulated using resting images. Finally, blood flow and pressure are modeled by solving the 3D Navier–Stokes equations.

### 5.1. HeartFlow^®^ FFR-CT: A Pioneering Non-Invasive Solution

**Figure 1:**
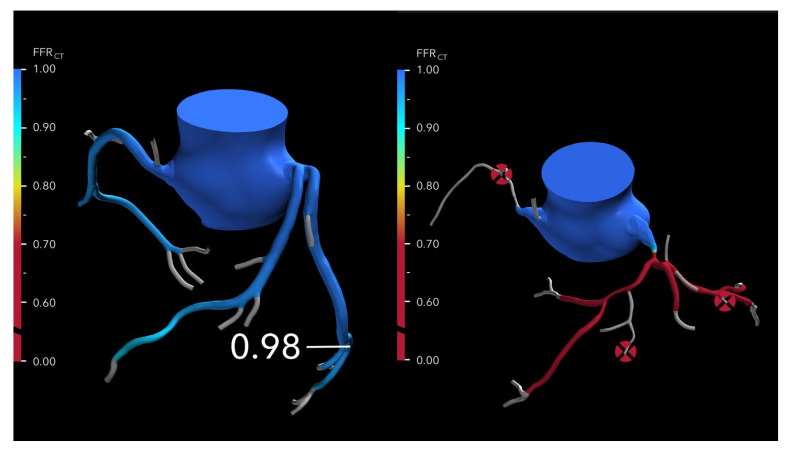
FFR-CT results are displayed using 3D-modeled representations of coronary arteries. (**Left**): A value above 0.80 is considered normal and is shown in blue. Borderline values ranging from 0.76 to 0.80 are depicted in orange and yellow. (**Right**): Values of 0.75 or below are classified as abnormal and marked in red. Areas of complete occlusion are indicated with an “X” symbol.

HeartFlow^®^ FFR-CT is the non-invasive technology for assessing the physiological significance of CAD most widely adopted in the United States. It remains the only solution with FDA clearance, CE marking, and NICE endorsement for the functional evaluation of coronary lesions. This innovative approach leverages CCTA and applies advanced computational fluid dynamics (CFD) to simulate blood flow and pressure within the coronary arteries. To perform the analysis, CCTA data is securely transferred to HeartFlow^®^’s centralized core laboratory, where powerful off-site supercomputers process the information. The CFD model reconstructs a patient-specific 3D model of coronary anatomy, calculates pressure gradients, and generates an FFR-CT map that identifies flow-limiting stenoses (Figure 1). The entire process typically takes between one and four hours, providing clinicians with a detailed, non-invasive assessment of coronary physiology.

### 5.2. The Evolving Role of On-Site Machine Learning-Based FFR-CT in Clinical Practice

On-site machine learning-based FFR-CT (FFR-CT_ML)_ represents a major advancement in the noninvasive physiologic assessment of coronary artery disease. Traditional FFR-CT solutions, such as HeartFlow^®^’s CFD-based platform, require an off-site supercomputing infrastructure. This approach poses limitations related to processing time, cost, and scalability, making it less accessible in time-sensitive or resource-limited settings. Recent developments in artificial intelligence have enabled on-site FFR-CT computation using deep learning models trained on synthetic coronary anatomies and corresponding hemodynamic data. These models rapidly approximate the complex nonlinear relationship between coronary anatomy and blood flow, bypassing the need for computational fluid dynamics. By operating on standard workstations, FFR-CT_ML_ enables faster reporting with a lower computational burden.

A recent meta-analysis evaluated the diagnostic performance of FFR-CT_ML_ [13]. A total of 23 studies on FFR-CT_CFD_ and 18 studies on FFR-CT_ML_ were included. The data encompassed 2501 patients and 3764 vessels in the CFD group, and 1323 patients and 4194 vessels in the ML group. The results demonstrated that FFR-CT_ML_ and FFR-CT_CFD_ had comparable specificity and AUC at both the patient (Z = 0.59, *p* = 0.55; AUC *p* = 0.5) and vessel (Z = 0.94, *p* = 0.34; AUC *p* = 0.74) levels. The diagnostic odds ratio was also similar at the vessel level (Z = 0.7, *p* = 0.48). However, FFR-CT_ML_ showed significantly lower sensitivity than FFR-CT_CFD_ at both the patient (Z = 3.85, *p* = 0.0001) and vessel (Z = 2.05, *p* = 0.04) levels. While the sensitivity of CFD-based FFR-CT may be superior, the specificity and area under the receiver operating curve, more relevant for clinical decision-making, are largely equivalent in the ML and CFD models. Importantly, on-site FFR-CT_ML_ can optimize workflow, reduce turnaround time, and improve cost-effectiveness, which may be particularly impactful in high-volume or under-resourced healthcare systems. Given its favorable diagnostic profile, operational advantages, and potential to enhance equitable access to CAD diagnostics, on-site FFR-CT_ML_ is poised to become an integral component of contemporary cardiac imaging strategies. Cleerly ISCHEMIA is an FDA-cleared automated machine learning-based decision-support tool used for interpreting CCTA images to estimate the likelihood of ischemia in individual vessels.

## 6. Real-World Evidence and Clinical Validation of FFR-CT

FFR_CT_ has been evaluated in several large-scale multicenter trials. The DISCOVER-FLOW study was the first to evaluate the diagnostic performance of FFR-CT, using a first-generation algorithm in patients with suspected or known CAD [14]. Involving 103 patients and 159 vessels, the study compared non-invasive FFR-CT with traditional coronary CTA, using invasive FFR as the gold standard. An FFR threshold of ≤0.80 and a CTA stenosis threshold of ≥50% were applied to define ischemia-causing lesions. FFR-CT demonstrated superior diagnostic accuracy (84% vs. 59%) and specificity (82% vs. 40%) compared to CTA, with similar sensitivity (88% vs. 91%). The area under the receiver operating characteristic (ROC) curve was significantly higher for FFR-CT (0.90) than for CTA (0.75, *p* = 0.001), indicating better overall diagnostic discrimination. FFR-CT also showed strong correlation with invasive FFR (r = 0.717, *p* < 0.001), with only a minor systematic underestimation. These findings established FFR-CT as a highly accurate, non-invasive alternative for the identification of functionally significant coronary stenoses.

The DeFACTO study evaluated the diagnostic performance of FFR-CT combined with CCTA in 252 stable patients with suspected or known CAD [15]. Conducted across 17 centers in five countries, the study compared non-invasive FFR-CT against invasive FFR as the gold standard. While the study narrowly missed its pre-specified accuracy target, FFR-CT plus CCTA demonstrated improved diagnostic accuracy (73%) compared to CCTA alone (64%). Sensitivity for detecting ischemia was significantly higher with FFR-CT (90% vs. 84%), while specificity remained modest (54%). Importantly, overall diagnostic discrimination improved, with an area under the receiver operating characteristic curve (AUC) of 0.81 for FFR-CT compared to 0.68 for CCTA alone (*p* < 0.001). In patients with intermediate stenosis (30–70%), FFR-CT also showed markedly improved sensitivity (82% vs. 37%). These findings highlight FFR-CT’s potential to enhance the physiological assessment of CAD beyond anatomical imaging alone.

The NXT study evaluated 254 patients with suspected stable coronary artery disease to assess the diagnostic performance of noninvasive FFR-CT [16]. Using invasive FFR ≤ 0.80 as the reference standard, FFR-CT demonstrated significantly improved diagnostic accuracy and discrimination compared to coronary CTA alone. The area under the curve (AUC) was 0.90 for FFR-CT versus 0.81 for CTA (*p* = 0.0008). While sensitivity was similar (86% for FFR-CT vs. 94% for CTA), specificity improved markedly (79% vs. 34%). These findings support FFR-CT as a superior noninvasive method for identifying ischemia-causing lesions.

A sub-analysis of the PACIFIC trial assessed 208 patients with suspected coronary artery disease, comparing the diagnostic performance of FFR-CT, coronary CTA, 99mTc-tetrofosmin SPECT, and ^15^O-H_2_O PET against invasive FFR as the reference [17]. On a per-vessel basis, FFR-CT demonstrated the highest AUC (0.94), outperforming CTA (0.83), SPECT (0.70), and PET (0.87). On a per-patient level, FFR-CT and PET had similar AUCs (0.92 vs. 0.91), both higher than CTA (0.81) and SPECT (0.75). However, on an intention-to-diagnose analysis, PET had superior performance due to FFR-CT’s limited evaluability—17% of vessels and 25% of patients were not analyzable. This limitation of FFR-CT, the requirement of high-quality image data, is also evident in prior studies, where 11% and 13% of examinations were non-evaluable, due to artifacts, in the DeFACTO and NXT trials, respectively. These findings underscore PET’s high diagnostic accuracy and suggest that while FFR-CT offers excellent functional assessment, its image quality dependency can affect real-world diagnostic utility compared to PET.

The PLATFORM trial was a multicenter study enrolling 584 patients with new-onset chest pain, comparing the usual care to a diagnostic strategy incorporating CCTA and selective fractional flow reserve derived from CTA (FFR-CT) [18]. Patients were assigned, based on the initial planned strategy, noninvasive testing or invasive coronary angiography (ICA), and then randomized to usual care or CTA plus FFR-CT. Among those for whom the initial plan was ICA, the CTA/FFR-CT strategy significantly reduced the number of patients undergoing ICA with no obstructive CAD, from 73% in usual care to 12% in the CTA/FFR-CT group. Additionally, 61% of invasive angiographies were avoided after CTA/FFR-CT results. Clinical event rates, including those for death and myocardial infarction, were low and similar between groups at one year. Moreover, the CTA/FFR-CT approach reduced costs by 33% in patients planned for invasive angiography, demonstrating that FFR-CT effectively acts as a gatekeeper to ICA, reducing the number of unnecessary invasive procedures without compromising patient outcomes.

The ADVANCE registry was a large prospective study of 5083 patients with suspected CAD, across 38 international centers [19]. It evaluated the real-world impact of incorporating FFR-CT into clinical decision-making. The study found that adding FFR-CT to standard CCTA led to changes in treatment strategy in nearly 67% of patients. Among those undergoing ICA, patients with an FFR-CT ≤ 0.80 had a significantly lower incidence of non-obstructive CAD (14.4%) compared to those with an FFR-CT > 0.80 (43.8%). Furthermore, 72.3% of patients with FFRCT ≤ 0.80 who underwent ICA proceeded to revascularization. At the 1-year follow-up, revascularization rates were substantially higher in the FFR-CT ≤ 0.80 group (38.4%) than in those with higher FFRCT values (5.6%). The findings highlight that FFR-CT effectively guides treatment by identifying patients likely to benefit from revascularization while reducing the number of unnecessary invasive procedures.

The SYNTAX III Revolution trial investigated how incorporating FFR-CT influences treatment decisions in patients with complex coronary artery disease, specifically those with left main or three-vessel disease [20]. The study randomized 223 patients to two heart teams, each comprising an interventional cardiologist, cardiac surgeon, and radiologist, who initially assessed patients using either coronary CTA or ICA. Each team calculated the anatomic SYNTAX score and recommended treatment strategies including coronary artery bypass grafting (CABG), percutaneous coronary intervention (PCI), and equipoise coronary artery bypass grafting–percutaneous coronary intervention. After reviewing the initial decisions, teams integrated FFR-CT data to reassess treatment plans. The addition of FFR-CT led to a change in overall treatment strategy in 7% of cases and altered vessel selection for revascularization in 12%. Furthermore, FFR-CT reclassified 14–16% of patients to a lower SYNTAX score category. These findings underscore the value of FFR-CT as a noninvasive functional tool that refines clinical decision-making and procedural planning in complex coronary disease.

A recent study evaluated the impact of FFR-CT on postprocedural myocardial injury (PMI) and long-term outcomes in revascularized OCAD patients, using data from a 2013–2021 tertiary hospital registry [21]. Among 559 revascularized patients (386 PCI, 166 CABG), FFR-CT was performed in 45.1%. Over 4.4 ± 2.2 years of follow-up, major adverse cardiovascular events (MACE) occurred in 24.5% of patients. FFR-CT use was associated with significantly lower all-cause mortality (HR 0.476, 95% CI 0.230–0.985, *p* = 0.046) and a non-significant trend toward reduced MACE (HR 0.736, 95% CI 0.513–1.055, *p* = 0.095). Subgroup analysis in PCI and CABG cohorts showed no significant MACE reduction individually. Among 477 patients with cardiac troponin data, 42.5% had PMI, with a lower PMI incidence in CABG patients undergoing FFR-CT (5.3% vs. 15.6%, *p* = 0.044), though this was not significant in adjusted models. These findings suggest that FFR-CT may improve long-term outcomes, particularly survival, independent of procedural myocardial injury.

One meta-analysis, encompassing 43 studies and over 7000 vessels from 5236 patients, provides the most extensive evaluation to date of the diagnostic performance and clinical applicability of FFR-CT compared to invasive FFR [22]. This analysis found a moderate correlation between FFR-CT and invasive FFR (Spearman coefficient 0.67), with the level of agreement increasing in vessels with higher FFR values, particularly those with an FFR-CT > 0.90. Overall, FFR-CT demonstrated robust diagnostic accuracy, sensitivity, and specificity (82.2%, 80.9%, and 83.1%, respectively). The diagnostic performance of FFR-CT varied by value range: values > 0.90 had a 93% likelihood of ruling out hemodynamically significant CAD, while an FFR-CT < 0.49 had nearly 100% accuracy in identifying physiologically significant lesions. This suggests that at either end of the FFR-CT spectrum, the modality performs exceptionally well and can guide confident clinical decision-making. In contrast, diagnostic uncertainty increases within the intermediate range or “gray zone” of 0.75–0.80, in which the accuracy drops significantly, potentially requiring additional testing before deciding on revascularization or deferral. Notably, FFR-CT readings on average were only 0.01 lower than invasive FFR, indicating minimal systematic bias. This highlights the utility of raw FFR-CT values for guiding decisions, rather than relying solely on dichotomous classification (i.e., ≤0.80 vs. >0.80). The clinical implications of these findings are significant. When the FFR-CT is >0.90, ICA may be safely deferred in most patients, helping to reduce unnecessary procedures, associated complications, and healthcare costs. Conversely, lesions with an FFR-CT < 0.49 are highly likely to benefit from revascularization. As a result, FFR-CT may serve as an effective gatekeeper for ICA, offering a valuable adjunct to anatomical imaging from CTA by adding a functional dimension. This aligns with growing interest in noninvasive “one-stop shop” approaches for evaluating chest pain and suspected CAD.

## 7. When and How to Use FFR-CT: Guidance for Coronary CTA Interpretation and Reporting

**Figure 2:**
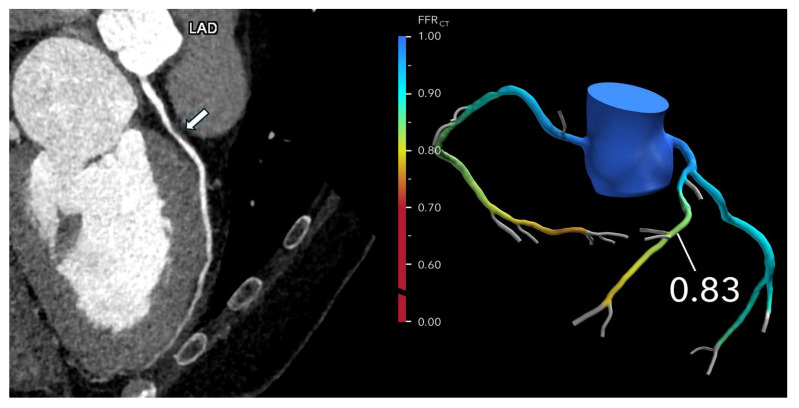
Optimizing treatment decisions with FFR-CT: (**Left**): A curved MPR coronary CTA image of the LAD in a 62-year-old man with atypical chest pain reveals severe stenosis (>70% diameter reduction) in the proximal LAD (arrow). (**Right**): The FFR-CT image demonstrates a value of 0.83 measured 2 cm distal to the lesion, indicating normal flow. As a result, invasive coronary angiography was deferred, and the patient was managed with medical therapy.

**Figure 3:**
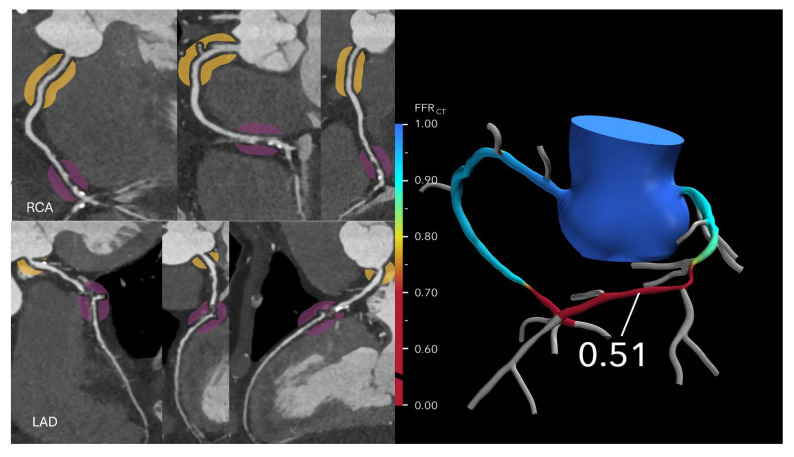
Optimizing treatment decisions with FFR-CT: (**Left**): A curved MPR coronary CTA image in a 64-year-old man with stable chest pain reveals severe stenoses in the mid left anterior descending (LAD) artery and distal right coronary artery. (**Right**): The corresponding FFRCT image shows a value of 0.51 measured 2 cm distal to the lesion, indicating severely reduced flow. Based on these findings, the patient proceeded to invasive coronary angiography followed by intervention.

FFR-CT is particularly valuable in evaluating patients with intermediate-risk coronary anatomy, typically characterized by one or more lesions causing 30–69% stenosis, or ≥70% stenosis in vessels other than the left main or proximal left anterior descending (LAD) artery. In these cases, FFR-CT helps determine the need for ICA versus optimal medical therapy (OMT). If the FFR-CT is >0.80, the likelihood of hemodynamically significant CAD is low, and patients can generally be managed safely with OMT alone, potentially avoiding unnecessary ICA or revascularization (Figure 2 and Figure 3).

FFR-CT is generally not indicated in low-risk anatomy, such as patients with normal CCTA or stenosis < 30%, for whom OMT is the standard approach. In high-risk anatomy, including ≥50% left main stenosis, ≥70% proximal LAD stenosis, or three-vessel disease, patients are typically referred directly to ICA due to the high pre-test probability of significant disease. In the “gray zone” (0.75–0.80) management should be individualized based on lesion characteristics such as location (e.g., LAD vs. non-LAD vessels), lesion segment (proximal vs. distal), number of affected vessels, total plaque burden, and high-risk plaque features (e.g., low-attenuation plaque, napkin-ring sign, and positive remodeling). In the absence of high-risk features or symptoms, many patients in this range may be safely managed with OMT and close clinical follow-up. One commonly used approach includes a 3-month trial of OMT, with ICA reserved for persistent or worsening symptoms.

**Figure 4:**
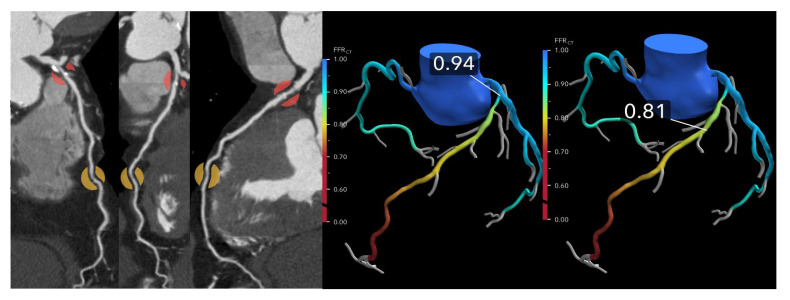
Lesion-specific ischemia: (**Left**): A curved multiplanar reformatted coronary CTA image in a 60-year-old man reveals moderate stenosis in the anterior left anterior descending (LAD) artery. (**Right**): The corresponding FFR-CT image shows an FFRCT value of 0.81 measured 2 cm distal to the lesion. The difference between the proximal and distal FFR-CT values across the lesion is 0.13, which exceeds the abnormal threshold of 0.12, confirming hemodynamically significant ischemia.

A metric to enhance physiologic interpretation of FFR-CT is the ΔFFR-CT, defined as the difference in FFR-CT values proximal and distal to a coronary lesion. In a study involving 73 vessels from 50 patients who underwent CCTA, FFR-CT, and invasive FFR, a ΔFFR-CT ≥ 0.12 strongly predicted ischemia (invasive FFR ≤ 0.80), with an odds ratio of 10.2 [23] (Figure 4). ΔFFR-CT demonstrated superior diagnostic accuracy (AUC 0.86) compared to diameter stenosis (AUC 0.66) and other FFR-CT metrics. Its integration significantly improved ischemia classification and reclassification, suggesting ΔFFR-CT may serve as a valuable adjunct, particularly in intermediate lesions or diagnostic uncertainty.

The location of FFR-CT measurement critically impacts diagnostic accuracy. Lesion-specific ischemia is best evaluated 1–2 cm distal to the stenosis rather than at the terminal vessel, where physiologic pressure naturally declines, even in the absence of disease, potentially leading to the overestimation of ischemia [24]. Thus, terminal-vessel measurements should be avoided. Distal-to-lesion FFR-CT measurement improves concordance with invasive FFR, enhances diagnostic precision, and may reduce unnecessary invasive testing.

The “gray zone” of FFR-CT values between 0.75 and 0.80 remains diagnostically challenging, as lesions in this range are ischemic on invasive FFR in approximately 50–60% of cases. To address this, a structured clinical algorithm is recommended:

### 7.1. Symptom Assessment

Prioritize patient-reported symptoms (frequency, exertional pattern, and response to anti-anginal therapy). Persistent angina or functional limitation may justify more aggressive investigation.

### 7.2. Anatomic Context

Evaluate lesion location (e.g., LAD vs. non-LAD), segment (proximal vs. distal), and number of affected vessels. Proximal LAD lesions in particular warrant lower thresholds for further testing or ICA.

### 7.3. Plaque Morphology

Use high-risk plaque features on CCTA, such as low-attenuation plaque, positive remodeling, napkin-ring sign, and spotty calcification, to stratify risk. The presence of ≥2 high-risk features favors early invasive evaluation.

### 7.4. Physiologic Adjuncts

Consider using ΔFFR-CT, defined as the difference in FFR-CT values proximal and distal to a coronary lesion. ΔFFR-CT may thus refine decision-making, especially when standard FFR-CT values are indeterminate.

### 7.5. Trial of Medical Therapy and Follow-Up

In the absence of high-risk features or disabling symptoms, initiate a 12-week trial of OMT with close outpatient follow-up. Repeated non-invasive testing (e.g., perfusion imaging or stress echo) or ICA should be reserved for symptom progression or diagnostic uncertainty.

This structured approach reinforces the utility of FFR-CT in intermediate-risk CAD while acknowledging its limitations as a standalone tool in borderline cases. It enables clinicians to combine physiologic, anatomic, and clinical factors in making safe and individualized decisions, thereby preserving the core benefit of FFR-CT, namely, reducing the incidence of unnecessary invasive testing without compromising diagnostic rigor.

## 8. Understanding the Limitations of the FFR-CT Technology

Accurate FFR-CT analysis relies on high-quality coronary CTA, requiring precise lumen segmentation and myocardial mass estimation; both are dependent on optimal spatial resolution and complete myocardial coverage. Image-degrading factors such as motion artifacts, calcium blooming, misalignment, noise, and poor contrast can obscure lumen boundaries or exclude sections of the myocardium from the field of view. Due to these limitations, 10–25% of studies are rejected and deemed unsuitable for FFR-CT analysis [16,18]. In approximately 80% of disqualified cases, motion artifacts were the main reason for inadequate image quality [25]. Additionally, FFR-CT is currently not feasible in patients who have undergone coronary stenting or coronary artery bypass grafting (CABG).

Coronary artery calcification (CAC) significantly affects the diagnostic accuracy of both CCTA FFR-CT. A recent meta-analysis evaluated ten studies comparing FFR-CT and CCTA performance across different coronary artery calcium score categories, using invasive FFR as the reference standard [26]. The results showed that FFR-CT consistently outperformed CCTA in detecting hemodynamically significant stenoses at low to moderate calcium levels (CACS < 400), with notably higher area under the curve (AUC) values, both per-patient and per-vessel. For example, in patients with a CACS < 100, FFR-CT AUC reached 0.9 versus 0.32 for CCTA, while in CACS 100–399, FFR-CT maintained superior accuracy. The superiority of FFR-CT at lower calcium burdens is attributed to its computational fluid dynamics (CFD) approach, which integrates global coronary anatomy and physiological flow models, reducing the impact of the calcification-related artifacts that limit CCTA’s lumen assessment. However, in patients with severe calcification (CACS ≥ 400), FFR-CT’s diagnostic advantage diminishes, with specificity decreasing and AUC values declining. Further research is needed to establish a clear CACS threshold guiding its clinical application.

Despite its diagnostic potential, the dependency of FFR-CT on high-quality image acquisition remains a critical limitation. In real-world practice, non-diagnostic scan rates ranging from 10–25% raise valid concerns about reproducibility, especially in high-volume centers managing patients with common comorbidities or suboptimal scan conditions. These failure rates can undermine diagnostic confidence, delay clinical decision-making, and reduce overall workflow efficiency. Until improvements in scanner technology, acquisition protocols, and motion correction algorithms become more widely adopted, the integration of FFR-CT into routine clinical workflows will need to be approached cautiously, with a realistic understanding of its current technical constraints. Overall, FFR-CT shows promise in improving non-invasive evaluation of CAD, but optimizing its robustness across diverse clinical scenarios remains an important area for future innovation.

## 9. The Cost-Effectiveness and Economic Impact of FFR-CT in Clinical Practice

The cost of FFR-CT also presents a barrier to its routine use, especially in non-invasive diagnostic pathways. The Centers for Medicare & Medicaid Services (CMS) have set national payment rates for the HeartFlow^®^ FFR-CT at $1017 for hospital outpatient settings and $839 for physician office settings. These rates are established under the Hospital Outpatient Prospective Payment System (OPPS) and the Physician Fee Schedule (PFS), respectively.

The PLATFORM study showed that using FFR-CT in evaluating stable patients with chest pain reduced healthcare costs [27]. The study evaluated 584 patients and found that using FFR-CT reduced costs by 32% in the invasive testing group ($7343 vs. $10,734; *p* < 0.0001) and improved quality of life in the noninvasive group (Seattle Angina Questionnaire score increase: 19.5 vs. 11.4; *p* = 0.003), compared to usual care. In the FORECAST trial (*n* = 1400), an FFR-CT-guided strategy showed no significant difference in total cardiac costs compared to standard care (£114 higher; *p* = 0.10) but significantly reduced invasive coronary angiography rates (19% vs. 25%; *p* = 0.01), suggesting potential economic efficiency attained by avoiding unnecessary invasive procedures [28].

However, while early trials suggest the potential for cost containment, these findings have limited generalizability to broader, real-world populations. The upfront cost of FFR-CT, over $1000 per analysis, remains financially prohibitive for many institutions, especially those in low- and middle-income countries or under tight budgetary constraints. Furthermore, reimbursement pathways for FFR-CT vary widely across health systems, often lacking clarity or consistency, which can deter adoption. Economic modeling studies have attempted to project long-term cost-effectiveness based on reduced downstream testing and improved care triage, but real-world validation of these models is still lacking. To facilitate broader implementation, future studies must evaluate FFR-CT’s value in diverse clinical settings, incorporating heterogeneous populations and real-world cost data. Additionally, health economic strategies, such as bundled payments, value-based reimbursement models, or integration into clinical decision-support tools, may help align FFR-CT adoption with sustainable cost structures. Without such considerations, the widespread integration of FFR-CT into routine practice may remain limited despite its clinical promise.

## 10. Looking Ahead: Unlocking the Future Potential of FFR-CT

### 10.1. Evaluation of Coronary Artery Anomalies

**Figure 5:**
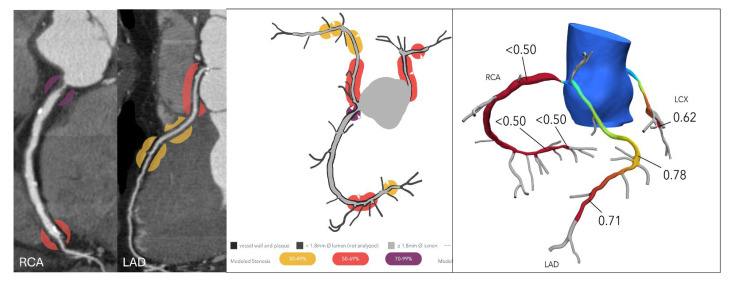
Anomalous aortic origin of the left coronary artery. (**Left**): Curved multiplanar reformatted coronary CT angiography (CTA) images in a 65-year-old male patient demonstrate severe stenosis at the ostium of the right coronary artery (RCA) and moderate stenosis at the ostium of the left anterior descending (LAD) artery, both originating from the right coronary cusp. (**Right**): Corresponding fractional flow reserve computed tomography (FFRCT) images show a value of 0.50 at 2 cm distal to the RCA ostial lesion, indicating significantly reduced flow. The mid LAD displays a value of 0.78, and the distal LAD shows a value of 0.71, suggesting a high likelihood of hemodynamic significance. Based on these findings, the patient proceeded to invasive coronary angiography and subsequent intervention for both lesions.

Anomalous aortic origin of a coronary artery (AAOCA), particularly with an interarterial course, is a known risk factor for sudden cardiac death, especially among young individuals. Traditional imaging often struggles to determine the functional significance of such anomalies. FFR-CT has emerged as a promising tool that provides both anatomical and physiological insight in a non-invasive manner. In the ANOCOR registry, 62 patients with AAOCA underwent FFR-CT analysis, revealing that FFR-CT values were significantly lower in those with key morphological markers of an intramural course, including a takeoff angle < 30°, luminal narrowing >50%, and eccentricity > 1.5. A proximal FFR-CT cutoff of ≤0.83 demonstrated high sensitivity (96%) and specificity (100%) for identifying intramural pathways [29]. These findings are notable, as the diagnostic threshold for AAOCA appears to be higher than the conventional ≤0.80 used in atherosclerotic disease. While FFR-CT reflects only the fixed anatomical component of AAOCA at rest, it may still aid in risk stratification, especially when considering dynamic changes during exertion. Complementing this, a separate study involving 94 patients with an interarterial anomalous right coronary artery from the left coronary sinus found that anatomical features such as slit-like ostium, low takeoff level, and presence of an intramural course were significantly associated with abnormal FFR-CT values (≤0.80) [30] (Figure 5). These anatomical predictors also correlated with a higher prevalence of both typical and atypical angina, highlighting FFR-CT’s potential to gauge clinical relevance and symptom burden in AAOCA.

### 10.2. Plaque Characterization

Coronary computed tomography angiography has become the leading noninvasive tool for evaluating both calcified and high-risk coronary plaque features. Beyond identifying luminal narrowing, CCTA provides critical insight into plaque composition, including low-attenuation plaque (LAP), positive remodeling, and the overall plaque burden features collectively termed high-risk plaque (HRP) [31]. These advanced markers not only help refine risk assessment for CAD but also enhance the ability to predict ischemia and acute coronary syndromes.

Recent studies have demonstrated that total plaque volume and the presence of HRP features are more closely associated with ischemia, as measured by fractional flow reserve, whether invasive FFR or computed tomography-derived (FFR-CT), than stenosis severity alone. Specifically, LAP has emerged as a strong and independent predictor of ischemia. In fact, patients with two or more HRP features on CCTA have more than a threefold increased likelihood of ischemia on FFR-CT compared to those with only one HRP marker [32]. Together, plaque characterization with CCTA and physiological assessment with FFR-CT offer a comprehensive, noninvasive approach to understanding coronary disease and guiding patient management.

## 11. Myocardial Infarction and Ischemia in the Absence of Obstructive Coronary Artery Disease

Myocardial infarction with non-obstructive coronary arteries (MINOCA) and ischemia with non-obstructive coronary arteries (INOCA) represent distinct clinical conditions within the spectrum of ischemic heart disease, occurring in patients without significant coronary artery obstruction. MINOCA is characterized by elevated cardiac biomarkers indicative of myocardial infarction, despite angiographic evidence of minimal or no coronary stenosis. Its potential causes are diverse and include plaque disruption, spontaneous coronary artery dissection (SCAD), Takotsubo syndrome, myocarditis, coronary vasospasm, microvascular dysfunction, and supply–demand mismatch [33]. In a recent study, females aged 70 years or younger with myocardial infarction with MINOCA experienced a significantly higher incidence of major adverse events compared to both their male counterparts and females with myocardial infarction with obstructive coronary arteries (MIOCA). This difference is likely attributable to the distinct underlying pathophysiological mechanisms of ischemia in MINOCA, which may include coronary microvascular dysfunction, vasospasm, or spontaneous coronary artery dissection, rather than the atherosclerotic plaque rupture typically seen in MIOCA. These findings highlight the need for tailored diagnostic and therapeutic strategies in younger women presenting with MINOCA [34].

In such cases, ICA and intravascular imaging may be necessary, yet CCTA and FFR-CT can serve as valuable tools to exclude obstructive disease and detect alternative etiologies such as SCAD. INOCA, on the other hand, is defined by ischemic symptoms without biomarker elevation and is frequently underdiagnosed or misattributed to non-cardiac conditions. Common underlying mechanisms include coronary vasospasm and microvascular dysfunction [35].

Beyond FFR-CT, the HeartFlow^®^ platform can compute the coronary volume-to-myocardial mass (V/M) ratio, offering additional insights into coronary flow capacity [36]. A low V/M ratio may suggest microvascular angina or myocardial supply–demand mismatch, helping to identify physiologic causes of ischemia in the absence of significant luminal narrowing [37,38]. Thus, in patients with MINOCA or INOCA, CCTA and FFR-CT, augmented by V/M analysis, offer a comprehensive, noninvasive approach to diagnosis and risk stratification.

In a prospective study of 153 patients with angina and nonobstructive coronary artery disease (ANOCA), 68 were found to have coronary microvascular dysfunction (CMD), and 22 of those exhibited high minimal microvascular resistance (>470 Wood units) [39]. Patients with CMD had a significantly lower coronary flow reserve compared to controls (1.9 ± 0.38 vs. 3.2 ± 0.81; *p* < 0.001). A strong inverse correlation was observed between lumen volume and microvascular resistance (r = −0.59; 95% CI: −0.45 to −0.71; *p* < 0.001). Those with CMD and high microvascular resistance had 40% smaller lumen volumes than controls (512.8 ± 130.3 mm^3^ vs. 853.2 ± 341.2 mm^3^; *p* < 0.001). Epicardial lumen volume, assessed by CCTA, was independently associated with microvascular resistance (*p* < 0.001). The ability of the CTA-derived lumen volume to predict high microvascular resistance demonstrated an area under the curve of 0.79 (95% CI: 0.69–0.88). These findings suggest CCTA may be valuable in the diagnostic workup of patients with CMD and ANOCA.

## 12. PCI and CABG Planning

One of the most promising advancements is the integration of FFR-CT into pre-procedural planning for PCI and coronary artery bypass grafting (CABG). The HeartFlow^®^ Planner tool enables cardiologists to simulate revascularization strategies by assessing lesion-specific ischemia, determining optimal stent size and placement, and performing virtual remodeling of the coronary lumen. This allows for a functional evaluation of how PCI might impact coronary flow, before any intervention is performed.

Early feasibility studies have shown that FFR-CT values obtained after virtual stenting closely correlate with post-PCI invasive FFR, highlighting the potential of the technique to predict procedural success [40,41]. In a prospective, multicenter observational study involving 120 patients, the HeartFlow^®^ Planner demonstrated high accuracy in matching both FFRINV and minimal stent area assessed via optical coherence tomography (OCT), even in complex scenarios involving diffuse disease and significant calcification [42]. In patients with tandem stenoses, the FFR-CT planner helps assess the added benefit of stenting each individual lesion. In cases of diffuse coronary disease, it supports optimizing the overall impact of the procedure while minimizing complications and limiting the total stent length used [43].

A recent patient-level meta-analysis of nine studies including 3336 coronary vessels (2760 patients) evaluated the prognostic value of post-PCI FFR across different coronary arteries [44]. The overall mean post-PCI FFR was 0.89 (95% CI: 0.87–0.90), with significantly lower values observed in left anterior descending (LAD) arteries compared to non-LAD vessels (0.86 vs. 0.93; *p* < 0.001). Lower post-PCI FFR was independently associated with increased risk of target vessel failure (TVF), primarily driven by target vessel revascularization. For every 0.10-unit decrease in FFR, the risk of TVF increased by 52%. However, predictive accuracy varied: post-PCI FFR had poor discrimination for TVF in the LAD (AUC: 0.52; 95% CI: 0.47–0.58) and moderate in non-LAD vessels (AUC: 0.66; 95% CI: 0.59–0.73; *p* = 0.005). These findings underscore the need for vessel-specific interpretation of post-PCI FFR and caution against applying uniform thresholds across all coronary territories.

Invasive coronary angiography has traditionally been the cornerstone for surgical planning in patients with complex multivessel CAD undergoing CABG. However, studies have shown that ICA alone may not reliably identify functionally significant stenoses, potentially leading to suboptimal graft targeting [45,46]. A major contributor to graft failure is competitive flow, particularly when grafts are placed to lesions that do not cause ischemia. This mechanism accounts for the failure of over 75% of internal thoracic artery (ITA) grafts in such cases [47]. Moreover, up to 25% of grafts do not result in improved regional myocardial perfusion post-CABG [48]. These findings underscore the importance of incorporating functional assessment into the preoperative evaluation to guide more effective and durable surgical revascularization strategies.

The role of FFR-CT as a non-invasive tool for identifying suitable CABG candidates and guiding surgical planning is increasingly recognized and supported by current clinical guidelines, based on evidence from multiple studies [49,50,51,52]. The first successful CABG procedure guided exclusively by FFR-CT has been recently documented [49], where post-operative FFR-CT assessments were also used to confirm functional improvement. Furthermore, the ongoing FASTTRACK CABG trial aims to evaluate the feasibility and reliability of planning CABG primarily using CCTA and FFR-CT, independent of invasive coronary angiography, potentially establishing a new paradigm in surgical decision-making [53]. These findings support the growing role of FFR-CT not only in diagnosis but also in guiding and optimizing therapeutic strategies, offering a noninvasive, patient-specific approach to coronary revascularization planning.

## 13. Filtering the Flow: FFR-CT as the New Gatekeeper to the Cath Lab

The main benefit of FFR-CT, demonstrated across multiple studies, is its ability to reduce unnecessary ICA [18,19,28]. In the PLATFORM trial, researchers found that an FFRCT-guided approach led to fewer referrals for cardiac catheterization, and patients who did undergo invasive coronary angiography were more likely to have obstructive CAD [18]. The ADVANCE study results demonstrated that FFR-CT led to a change in patient classification and influenced treatment plans in 66.9% of cases [19]. Additionally, among patients with an FFR-CT value of ≤0.80, subsequent invasive coronary angiography revealed a significantly higher likelihood of the identification of obstructive disease requiring revascularization [19].

The FORECAST trial was the first randomized study to evaluate the effectiveness of using CCTA combined with selective FFR-CT in patients with stable chest pain [28]. While overall medical costs were similar, the study found that patients assessed with FFR-CT were 22% less likely to undergo ICA. Moreover, when ICA was performed, those in the FFR-CT group had a 52% lower chance of having no obstructive coronary disease identified. These findings suggest that FFR-CT can help avoid unnecessary invasive procedures, while delivering clinical outcomes and quality of life comparable to traditional diagnostic pathways.

The PRECISE trial enrolled 2103 stable patients with suspected CAD across 65 sites, comparing a precision strategy (PS) to the usual testing (UT) [54]. The PS used the PROMISE minimal risk score to defer testing in 20.1% of low-risk patients, while others underwent CCTA with selective FFR-CT. The UT group received standard care, including stress testing or ICA.

At one year, the PS significantly improved clinical efficiency by reducing catheterizations without obstructive CAD from 10.2% in UT to 2.6% (hazard ratio [HR] 0.24; 95% CI, 0.16–0.36). The composite safety outcome of death or nonfatal myocardial infarction was similar between groups (HR 1.52; 95% CI, 0.73–3.15), with death rates of 0.5% vs. 0.7% and nonfatal MI rates of 1.2% vs. 0.5% for PS and UT, respectively. Notably, lipid-lowering (50.0% vs. 41.8%) and antiplatelet (35.7% vs. 27.1%) medication use rates were higher in the PS group (both *p* < 0.001).

These results demonstrate that FFR-CT, combined with risk stratification, effectively acts as a gatekeeper to the catheterization lab, reducing the number of unnecessary invasive procedures without compromising safety. The PRECISE trial supports adopting this approach for stable chest pain evaluation. However, it is important to recognize that most trials to date have enrolled highly selected patient populations, often excluding individuals with arrhythmias, high coronary calcium scores, obesity, renal dysfunction, prior revascularization, or other common comorbidities. These exclusions limit generalizability to real-world settings. Ongoing registry studies and advances in imaging technology are now aiming to address these gaps, and future research will be crucial to determine the effectiveness and scalability of FFR-CT across broader and more complex patient populations.

While FFR-CT demonstrates considerable promise in guiding the evaluation of stable chest pain, it is essential to contextualize its performance against other established and emerging diagnostic modalities. Positron emission tomography (PET), for example, offers superior spatial resolution and quantification of myocardial blood flow and coronary flow reserve, particularly useful in multivessel disease and microvascular dysfunction [55]. Positron emission tomography-derived myocardial flow reserve and invasive coronary flow reserve assess the combined effects of epicardial narrowing, diffuse atherosclerosis, and microvascular dysfunction on coronary blood flow. In contrast, fractional flow reserve primarily reflects the pressure gradient caused by focal and diffuse coronary artery disease. These measures are physiologically related but not interchangeable, with discordance observed in 30–40% of lesions.

Similarly, stress cardiac magnetic resonance imaging (CMR) provides high diagnostic accuracy and prognostic value, especially for identifying ischemia in patients with balanced or diffuse CAD. In a randomized trial of 918 patients with stable angina, CMR-guided care resulted in fewer index revascularizations than FFR-guided care (35.7% vs. 45.0%, *p* = 0.005). The primary outcome, major adverse cardiac events at one year, was similar between groups, meeting noninferiority criteria. These results support CMR as a safe and effective alternative [56].

Invasive pressure-derived indices such as instantaneous wave-free ratio (iFR) have also gained traction as an alternative to FFR, offering lesion-specific physiological assessment without the need for pharmacologic hyperemia. In a randomized trial of 2492 patients with CAD, instantaneous wave-free ratio (iFR)-guided revascularization was compared with FFR-guided revascularization [57]. At one year, major adverse cardiac events occurred in 6.8% of the iFR group and 7.0% of the FFR group, meeting noninferiority criteria. Procedural symptoms were significantly lower with iFR (3.1% vs. 30.8%, *p* < 0.001), and median procedure time was shorter (40.5 vs. 45.0 min, *p* = 0.001). These findings support iFR as a safe, efficient, and adenosine-free alternative to FFR in guiding coronary revascularization.

Hybrid diagnostic strategies combining anatomical and functional data, such as CCTA followed by PET or CMR in equivocal cases, can improve diagnostic accuracy and cost-effectiveness, particularly in complex or intermediate-risk patients. Thus, while FFR-CT offers non-invasive physiological assessment with good negative predictive value, it should be considered as part of a broader diagnostic toolbox rather than a standalone replacement. Tailoring test selection based on patient characteristics, availability, and clinical context remains critical to optimizing care.

## 14. Conclusions

Coronary computed tomography angiography and computed tomography-derived fractional flow reserve are rapidly redefining the landscape of CAD diagnosis and management, serving as both gatekeepers to the catheterization lab and catalysts for a more precise, noninvasive approach to modern cardiology. When used together, CCTA and FFR-CT deliver comprehensive insights into coronary anatomy, lesion-specific ischemia, high-risk plaque features, vessel wall stress, and volumetric metrics such as the vessel-to-myocardium ratio, none of which can be achieved through traditional noninvasive testing alone.

This integration of detailed anatomical and physiological data enables a more accurate diagnosis and risk stratification, reducing the number of unnecessary invasive coronary angiographies and allowing only truly indicated cases to proceed to the catheterization laboratory. Furthermore, emerging capabilities such as virtual PCI planning and functional SYNTAX scoring elevate these tools from a purely diagnostic function to therapeutic planning platforms. They assist in stent sizing and placement, and in predicting post-intervention flow, all from a noninvasive, pre-procedural standpoint.

Though limitations remain, particularly in patients with prior coronary stents or bypass surgery, the potential for CCTA and FFR-CT to replace invasive coronary angiography as the default diagnostic approach for stable coronary artery disease is becoming increasingly realistic. As image quality improves, costs decline, and computational power increases, the routine application of FFR-CT may become more accessible and widespread.
